# Inflammatory and Infectious Processes Serve as Links between Atrial Fibrillation and Alzheimer’s Disease

**DOI:** 10.3390/ijms21093226

**Published:** 2020-05-02

**Authors:** Gabriela Lopes Martins, Rita Carolina Figueiredo Duarte, Marat Alexandrovich Mukhamedyarov, András Palotás, Cláudia Natália Ferreira, Helton José Reis

**Affiliations:** 1 Instituto de Ciências Biológicas, Universidade Federal de Minas Gerais, BR-31270-901 Belo Horizonte, Brazil; gabrielalmartins@gmail.com (G.L.M.); ritinhaduarte@yahoo.com.br (R.C.F.D.); ferreiracn@gmail.com (C.N.F.); heltonjr@ufmg.br (H.J.R.); 2 Department of Physiology, Kazan State Medical University, R-420012 Kazan, Russia; maratm80@list.ru; 3 Asklepios-Med (Private Medical Practice and Research Center), H-6722 Szeged, Hungary; 4 Institute of Fundamental Medicine and Biology, Kazan Federal University, R-420008 Kazan, Russia

**Keywords:** Alzheimer’s disease, anti-inflammatory therapy, arrhythmia, atrial fibrillation, dementia, endothelial damage, infection, inflammation, microbiome, predictive biomarkers

## Abstract

Atrial fibrillation (AF) is one of the most prevalent forms of arrhythmia that carries an increased risk of stroke which, in turn, is strongly associated with cognitive decline. The majority of dementia cases are caused by Alzheimer’s disease (AD) with obscure pathogenesis. While the exact mechanisms are unknown, the role of inflammatory processes and infectious agents have recently been implicated in both AD and AF, suggesting a common link between these maladies. Here, we present the main shared pathways underlying arrhythmia and memory loss. The overlapping predictive biomarkers and emerging joint pharmacological approaches are also discussed.

## 1. Introduction

Atrial fibrillation (AF) is the most frequent type of cardiac arrhythmia. It has a prevalence of around 1%–2%, and its incidence significantly increases with age [1]. AF is associated with high morbidity and mortality due to its main complications, including heart failure (HF) and stroke [2], which are caused by significant alterations directly attributable to AF, such as hemo-dynamic changes, atrio-ventricular dys-synchrony, progressive mechanical dysfunction of the atria and ventricles, and thrombo-embolic events. In addition, several studies suggest a relationship between AF and cognitive decline, including Alzheimer’s disease (AD) and other forms of dementia [3,4,5,6].

Both AF and AD have risk factors in common, such as age, type 2 diabetes mellitus and cardio-vascular diseases [4]. In particular, heart failure was associated with dementia and AD in a cohort study involving 1301 subjects (mean age 83.3 ± 5.4 (HF) and 81.2 ± 4.8 (no HF), male 20% (HF) and 23% (no HF); hazard ratio (HR) 1.84, 95% confidence interval (CI) 1.35–2.51) [7]. Other epidemiological studies demonstrated the relationship between AF and AD. In a cohort of 3045 adults over 65 years old, followed for 14 years, AF was associated with a higher risk of all-cause dementia (HR 1.38, 95% CI 1.10–1.73) and possible or probable AD (HR 1.50, 95% CI 1.16–1.94) [8]. A population-based research that evaluated 2000 individuals from the Cardiovascular Risk Factors, Aging and Dementia (CAIDE) study associated AF in late life as an independent risk factor for dementia (HR 2.61, 95% CI 1.05–6.47, *p* = 0.039) and AD (HR 2.54, 95% CI 1.04–6.16, *p* = 0.040) [9]. In a cross-sectional study involving 784 subjects (mean age 77.5 years, 59.2% female), patients with AF had a two-fold higher prevalence of dementia than the control group (21.4% vs. 10.7%, *p* = 0.024), and AD was also more frequent among individuals with AF (12.6% vs. 7.3%, *p* = 0.046) [10]. Furthermore, a longitudinal study that followed 37,025 subjects for 5 years (mean age 60.6 years, 39% female) associated AF with all types of dementia, independently (odds ratio (OR) 1.44, *p* < 0.0001), and the highest risk for AD development occurred among younger individuals (≤70 years, OR 2.30, *p* = 0.001) [11].

Despite these results, there is little neuropathological evidence showing the association between AF and AD [3]. A cross-sectional study demonstrated an association between AF and a reduction in hippocampal volume, which is a neuropathological finding of AD, in stroke-free individuals with AF [12]. A study that evaluated the autopsies of 328 participants observed a higher prevalence of neuritic plaques and neuro-fibrillary tangles, which are neuropathological changes associated with AD, in individuals with permanent AF (relative risk (RR) 1.47, 95% CI 0.96–2.28) compared to non-AF subjects (RR 1.40, 95% CI 0.79–2.49) [13]. In contrast, it was also suggested in a study that HF and AF are associated with milder AD neuropathology [5].

Considering the evidence, there are some mechanisms proposed to relate these diseases. In persistent AF, there is a decrease in cardiac output which, in turn, can promote a state of chronic cerebral hypoperfusion and hypoxia. As a result, these factors can alter the blood–brain barrier (BBB) permeability, leading to the impaired clearance of β-amyloid peptide (βAP). Thus, it may lead to an accumulation of βAP in brain, which is the main pathologic hallmark of AD [3,14,15,16,17]. Moreover, the chronic reduction in cerebral perfusion that occurs in AF may be also related to local acidosis and a rise in the oxidative balance of the brain. These factors can change the functioning of tau protein, which can lead to hyperphosphorylation and the formation of tau oligomers, which are also important hallmarks of AD [16,18].

Although most studies have attempted to demonstrate that AF can lead to the development of AD, the reverse is also possible. In a retrospective cross-sectional study from a cohort of AD patients and age-matched controls, the myocardial function was examined. After echo-cardiographic analyses, the subjects with AD showed an anticipated diastolic dysfunction. Moreover, the expression of pathological forms of βAP was present not only in the brain of these patients but also in the heart [19]. Considering that diastolic dysfunction is a risk factor for AF, these results suggest that AD may lead to AF by the accumulation of βAP in the heart, suggesting that the relationship between these clinical conditions may be bi-directional [3]. However, further studies in this line of reasoning are necessary to evaluate this association.

Another important factor that could connect these two diseases is the inflammatory process that occurs in both. It is already known that in AF there is systemic inflammation and several studies have associated AF with inflammatory markers, reported both as a cause and as a consequence of this arrhythmia [20,21]. There is also clear evidence that in the brains of subjects with AD, the activation of inflammatory pathways occurs [22,23]. At first, it was believed that inflammation in AD occurred as a response to the pathophysiological events of the disease. More recently, there are growing reports that inflammatory processes may contribute to the pathogenesis of AD [24]. In addition, considering that inflammatory mechanisms may occur due to infectious processes, there is also evidence suggesting that some infectious diseases may be involved in the pathophysiology of AF [25] and AD [26] and can also be a link between both. In view of the above, this review aimed to examine the link between atrial fibrillation and Alzheimer’s disease, focusing on the inflammatory and infectious aspects that are related to both conditions as well as discussing the pharmacological approaches related to these diseases.

## 2. Inflammatory Mechanisms in AF and in AD, and Its Association between Both Diseases

Inflammation can be triggered by arrhythmogenic risk factors, such as systemic diseases (coronary artery disease, hypertension, and obesity), myocardial lesions (infarction and cardiac surgeries), and valvular diseases [21,27]. In this context, the increase in circulating inflammatory mediators may lead to the cardiac electrical and structural remodeling observed in AF; in this case, inflammation can be considered a cause of the disease. However, inflammatory processes also occur in response to AF, sustaining the disease by the same mechanism of remodeling. This has generated the expression “AF begets AF” [28].

The inflammatory response induced by AF is also related to the complications of the disease, such as thromboembolic events [28]. It is known that thrombogenesis occurs due to an endothelial injury/dysfunction followed by platelet/endothelial activation, with a consequent activation of the coagulation cascade. Endothelial injury can be induced by the turbulent and static flow in the atrium caused by AF [20]. This leads to an inflammatory response, with the infiltration of immune cells into the atrium; the decreased expression of endothelial nitric oxide synthase; and the increased expression of the von Willebrand factor, thrombin, thromboplastin, and protease-activated receptors. Monocytes and neutrophils that are partially activated by cytokines may also interact and activate platelets, evidencing, thus, the role of inflammation in the formation of thrombi in patients with AF [28]. It is important to consider that the left atrial appendage, which consists of a recess in the wall in the atrium, is the most common site of thrombus formation, since it is longer and has a narrow entrance, predisposing it to blood stasis [29].

In regard to AD, the mechanism of inflammation is also complex. There is evidence that βAP fibrils and soluble oligomers can bind with receptors expressed by microglia, promoting the activation of these cells. Once activated, there is a morphological change in microglia, which promotes βAP phagocytosis and releases different cytokines and inflammatory mediators in an attempt to prevent further damage caused by βAP deposits [23]. However, although microglia is an immune cell of the central nervous system (CNS) that performs neuroprotective actions in the brain, there is evidence that microglia can also be neurotoxic by killing or damaging neurons. This state possibly is promoted by a continuous microglial activation, leading to a “hyperactivity” of microglia, which do not become more capable of phagocytizing βAP deposits but keep the secretion of pro-inflammatory cytokines, contributing to neuronal death and thus to the neurodegeneration observed in AD [22].

The role of microglia has possibly been the most described, but the involvement of different patterns of CNS cells are also reported, such as astrocytes and others [24]. In this context, there is evidence that astrocytes surround βAP plaques in AD, becoming activated and secreting inflammatory mediators in same way as microglial cells [24].

These reports suggested that inflammatory responses are activated by the pathological stimuli in the brain, however, as mentioned above, there are studies proposing that inflammation can also be related to the pathogenesis of AD. In this way, it was suggested that a dysregulation of cytokines and chemokines in periphery may lead to the development of AD [22,30].

A possible factor that can contribute to this condition is the increased permeability of the BBB, which occurs naturally with aging [30,31]. In a cross-sectional study, it was observed that patients with AD presented an alteration in BBB permeability, which may affect passive and active transport processes [32]. It is worth mentioning that with aging there is the establishment of a chronic systemic inflammation due to the dysregulation of the immune system [33]. Thus, is possible to associate the exacerbated inflammatory state in the elderly with the alteration in BBB function [34].

Moreover, it was suggested that inflammatory mediators or even leukocytes from the blood could pass to the cerebrospinal fluid (CSF) in AD through the blood–cerebrospinal fluid barrier (BCSFB). In an exploratory study, the levels of different cytokines were measured in the serum and CSF of patients with AD, and the transport of these proteins from the serum to the CSF were related to a severe disruption in the BCSFB [30].

Given the above, is possible to consider that chronic conditions that promote the increase of circulating cytokines may be associated with the development of AD. It is not surprising that in situations considered to be risk factors for AD, such as diabetes mellitus and cardiovascular diseases, there are increased levels of inflammatory mediators [23]. Although AF was not suggested as a direct risk factor for AD, considering the high systemic inflammation that exists in this arrhythmia, it was suggested that individuals with AF may be more susceptible to a disruption in the BBB once inflammation increases the endothelial response to hypoxia [35]. With the altered BBB permeability, the passage of inflammatory molecules of serum to the CSF can also occur [16,17,30]. Thus, this can lead to cognitive decline and dementia, including AD [36].

### 2.1. Inflammatory Mediators Associated with Both AF and AD

Inflammatory markers—such as interleukin (IL)-1β, IL-6, tumor necrosis factor (TNF), IL-10, and others—have been associated with AF in several studies [21,37,38,39]. The same cytokines were also implicated in the pathophysiology of AD [23,40,41,42]. Given the mechanism proposed in the previous topic, it is possible to suggest that the elevated serum levels of inflammatory mediators in patients with AF may initiate a neuroinflammatory process by passing the BBB, which can lead to the development of AD. In Table 1, the major inflammatory molecules that are associated with both AF and AD are described.

### 2.2. Endothelial Damage Associated with Inflammation and Both AF and AD

Another situation that can connect AF and AD is endothelial dysfunction. Endothelial cells have a crucial role in the regulation of oxidative stress, vascular permeability, platelet aggregation, and the formation of thrombi, among other functions [55].

Several studies have shown that due to the alterations in hemodynamics occurring in AF, it may promote a dysfunction in the endothelium [4,21,56]. The altered electrical conduction in the atrium leads to a turbulent and static flow and, consequently, to an endothelial injury. From this, an inflammatory response is activated, promoting also trombogenesis, as mentioned above. The activation of the immune system leads to a decreased expression of endothelial nitric oxide synthase, which is important for the maintenance of normal endothelial function [20,55,57]. As the disease becomes chronic, the endothelial impairment promoted by AF may favor the maintenance of arrhythmia by the mechanism of vascular remodeling [58].

In this context, it was demonstrated in a meta-analysis that endothelial markers, as well as coagulation and fibrinolytic factors, were elevated in patients with AF in comparison to the control group [59]. Moreover, in another study, the endothelium dysfunction observed in patients with AF was worse in those with chronic forms of arrhythmia [58].

In parallel, evidence for endothelial dysfunction in AD has also been described, which suggests that this disease may present a vascular component in its pathophysiology [60]. In this way, it is possible to propose that the damage in endothelium promoted by AF can be related to AD, although the mechanisms involved in these processes are not well comprehended. As mentioned earlier, an increase in the permeability of the BBB in patients with AF may occur by an activation of the endothelial response to hypoxia. This can promote the release of inflammatory mediators, promoting damage in the endothelium cells that compose this barrier [35]. In addition, is important to mention that the impairment of endothelial function also occurs during aging, a condition that coexists in both diseases [61].

## 3. Infectious Agents Related to AF and AD as a Possible Link between Both Diseases

Considering the involvement of the inflammatory process in the physiopathology of AF and AD, and that chronic bacterial and viral infections are frequent causes of inflammation, there is evidence suggesting that infectious processes can also be related as a cause of both diseases [25,26].

In this context, the bacterial agent *Helicobacter pylori*, a gram-negative bacillus that can lead to gastritis and peptic ulcer disease, is also included. Montenero et al. [62] observed that serum positivity to *H. pylori* was highly correlated with AF. These results were related to the higher values of C reactive protein (CRP) found in patients with AF compared to controls. Furthermore, a strong correlation was observed between infection by *H. pylori* and patients with persistent AF, whose showed higher levels of seropositivity to the bacteria in comparison to patients with paroxysmal AF. In relation to AD, Kountouras et al. [63] found higher levels of *H. pylori* antibodies in the CSF of patients with AD in comparison to subjects without dementia. Huang et al. [64] also showed that subjects infected by *H. pylori* had a 1.6 times greater risk of developing AD in comparison to non-infected individuals. Moreover, infection by *H. pylori* was related to a reduction in cognition ability [65]. To explain the association between AD and the *H. pylori* infection, it was proposed that the access of *H. pylori* to the brain might occur by (1) an oral-nasal-olfactory pathway; (2) monocytes infected with this bacterium that produce higher levels of TNF, leading to a disruption in BBB; and (3) a rapid retrograde neural pathway from the gastrointestinal tract [66].

Given the above, is possible to suggest that infection by *H. pylori* may affect individuals with AF, especially in chronic states of arrhythmia, leading to an even stronger inflammatory response. Thus, it can increase the permeability of the BBB, favoring the passage of inflammatory molecules to the brain or even the infectious agent, which may be correlated directly with AD pathology. Further investigation is necessary to support this mechanism.

In line with these, viral agents, such as the herpes simplex virus (HSV)—which is a common cause of infection in the population and may cause watery blisters on the skin and mucous membranes—were also associated with AF and AD. In relation to the first, Chiang et al. [67] observed in a 3-year cohort study that patients with HSV showed a higher incidence of AF in comparison to those without this viral infection (*p* < 0.001). In addition, these authors found that HSV was independently associated with the risk of AF development (HR 1.39, 95% CI 1.2–1.6, *p* < 0.0001). In relation to AD, in a case-control study, a significant correlation was observed between HSV and dementia in older subjects (OR 2.250, *p* = 0.019) after 6.6 years of follow-up, suggesting that the virus could be related to the early development of AD [68]. Moreover, in a study involving 3432 subjects, an association was also demonstrated between the presence of anti-HSV immunoglobulin (Ig)M, which represents a reactivated infection, and the risk of developing AD [69]. Pathological findings also corroborate this relationship, as detected by Wozniak et al. [70]. These authors observed, in the brain autopsies of AD patients, a co-localization of HSV type 1 within amyloid plaques. Considering the above, it was suggested that HSV can access the brains of elderly subjects due to the impaired immune system response with age, together with the higher permeability of the BBB in older individuals [26].

In view of the above, it is also possible to link infection by HSV with the development of AF and AD by the same mechanism described above. On the basis of the foregoing, as preventive measures against the development of AF and AD, vaccines against these infectious agents could be reasonable options, although they are not yet available [25]. In addition, any clinical trial involving the use of vaccines for the prevention of these diseases would probably have to extend for many years to see any results of interest [71].

## 4. Pharmacological Approaches

### 4.1. Anti-Inflammatory Therapies for AF and AD

#### 4.1.1. Non-Steroidal Anti-Inflammatory Drugs and Corticosteroids

Currently, there are no anti-inflammatory therapies used in the clinical approach to AF and AD. The use of anti-inflammatory drugs, including non-steroidal anti-inflammatory drugs (NSAIDs) and corticosteroids, presents inconclusive results for both diseases. Regarding the use of NSAIDs in AF, besides not having demonstrated its benefits in patients with AF, the use of these drugs was associated with the risk of developing arrhythmia, as was demonstrated in a cohort study involving 8423 participants of the Rotterdam Study [72]. In this study, an association between the current use of NSAIDs and the risk of AF (HR 1.76, 95% CI 1.07–2.88) was observed. In a case-control study that enrolled 32,602 patients from northern Denmark, AF or a flutter were associated with the current use of non-selective NSAIDs as well as cyclooxygenase (COX)-2 inhibitors, with incidence rate ratios of 1.33 (95% CI 1.26–1.41) and 1.50 (95% CI 1.42–1.59), respectively [73]. A proposed mechanism to explain this is related to the inhibition of the cyclooxygenase (COX) in the kidneys by the NSAIDs. This may decrease potassium excretion in the distal nephron, leading to a fluctuation in the serum levels of this ion. As a consequence, it may cause a direct effect on the cell membrane, inducing arrhythmias [73,74,75].

With respect to the use of NSAIDs and AD, in a recent meta-analysis it was described that the current or previous use of these drugs was associated with a decreased risk of AD in comparison to non-users of NSAIDs. This association was verified including all types of NSAIDs except aspirin and acetaminophen [76]. In another meta-analysis, similar results were found, suggesting that non-aspirin NSAIDs may reduce the risk of AD, although further prospective studies are required to properly establish this association [77].

In relation to glucocorticoids and AF, in an experimental study, during treatment with prednisone the vulnerability to AF was reduced as well as the plasma levels of CRP [78]. In addition, in clinical studies, the use of corticosteroids appeared to reduce the incidence of postoperative AF [79,80] in addition to its recurrence after ablation therapy [81]. However, in a case-control study composed of 20,221 participants in northern Denmark with AF or a flutter, an association was observed between current therapy with glucocorticoids and an increased risk of these arrythmias (OR 1.92, 95% CI 1.79–2.06) [82]. In a cohort study, which included 7983 individuals of the Rotterdam Study, the use of high doses of corticosteroids was associated with a high risk of developing AF (OR 6.07, 95% CI 3.90–9.42) [83]. A possible reason for these results may be due to the cardiovascular side effects seen with the long-term use of glucocorticoids, such as hypertension, diabetes mellitus, and obesity, which are important risk factors of AF [84]. Considering these factors and the various adverse effects of glucocorticoids—such as hyperglycemia, susceptibility to infections, intestinal bleeding, and others—the benefits of its clinical use in AF may not be viable [28].

In relation to the use of corticosteroids and AD, in a study that evaluated the postmortem brains of subjects who had had neuritic plaques or neurofibrillary tangles, it was observed that those who used corticosteroids showed lower ratings and counts of these pathologic hallmarks in comparison to those who did not use these drugs [85]. However, in another study, the use of prednisone, for example, was not effective in the treatment of AD [86]. Moreover, there is evidence demonstrating that endogenous glucocorticoids may play a role in the pathophysiology of AD [87]. Based on this, more evidence is necessary to show if there are benefits in the use of corticosteroids for AD.

#### 4.1.2. Agents That Modulate Cytokines Signaling

The use of agents that can alter the signaling pathways of specific inflammatory mediators was suggested as an alternative in the pharmacological management of both AF and AD. For arrhythmia, for example, heat shock proteins were proposed (HSPs) as a molecular target. HSPs are important mediators of the inflammatory process, since they interfere with the TNF-signaling pathway, modulate the transforming growth factor (TGF)-β activity, and increase levels of IL-10 [28,88]. Mandal et al. [88] found higher intracellular levels of HSP70 in samples of atrial tissue in patients who did not develop AF after CABG in comparison with those who presented the arrhythmia. Hu et al. [89] studied HSP27, and lower levels of this marker were found in the peripheral blood samples from patients with AF compared to the controls. In addition, increased levels were detected in patients with paroxysmal AF compared to non-paroxysmal patients. Elevated HSP27 levels, in comparison to the baseline in individuals without arrhythmia, were also related to the maintenance of sinus rhythm in patients with AF who underwent catheter ablation therapy. Thus, it was suggested that these proteins may play a cardioprotective and anti-arrhythmic function in patients with AF [89].

With regard to AD, drugs that inhibit TNF signaling—such as anti-TNF antibodies (infliximab, for example), the immuno-suppressant rapamycin, and the immuno-modulator thalidomide—could be considered as potential therapies for the disease. In this context, in an experimental study, the intra-cerebral injection of infliximab in AD transgenic mice led to a temporary decrease in βAP and tau phosphorylation [90]. In addition, the AD mice model that received low doses of rapamycin for ten months exhibited an improvement in cognitive function and reduced levels of βAP and tau pathologies in the brain [91]. The same results were found for AD transgenic mice that received an analog of thalidomide for six weeks [92].

#### 4.1.3. Other Drugs That Have Anti-Inflammatory Properties

Besides these drugs, other classes of substances have been shown to play a potential role in the prevention of AF and AD and were considered anti-inflammatory because of their pleiotropic effects. These include the anti-hypertensive drugs, angiotensin converting enzyme inhibitors (ACEIs) and angiotensin-1 receptor blockers (ARBs); statins; colchicine; and poly-unsaturated fatty acids (PUFAs) [20,28].

ACEIs and ARBs, to date, have demonstrated a high therapeutic potential in AF, since they exhibit a role in the primary prevention of the disease in patients with different clinical situations, such as HF and hypertension [93]. In a meta-analysis, it was described that the use of renin-angiotensin-aldosterone system (RAAS) inhibitors in general—including ACEIs (perindopril, enalapril, ramipril, quinapril), ARBs (candesartan, valsartan, irbesartan), and mineralocorticoid receptor antagonists (eplerone, spironolactone)—significantly reduced the hospitalization of patients with HF (RR 0.89, 95% CI 0.82–0.97, *p* = 0.01), and improved their diastolic function [94]. Considering that HF is an important risk factor for AF, the treatment with RAAS inhibitors may be effective in the prevention of AF. Regarding AD, the use of both ACEIs and ARBs also showed benefits, as observed in a cohort study which followed subjects older than 75 years of age for a median of six years. Those that used ACEIs (benazepril, captopril, enalapril, fosinopril, lisinopril, moexipril, perindopril, quinapril, ramipril, trandolapril) and ARBs (candesartan, eprosartan, irbesartan, losartan, telmisartan, valsartan), as well as diuretics (amiloride, bumetanide, chlorthalidone, chlorothiazide, furosemide, hydrochlorothiazide, indapamide, metolazone, methylchlorothiazide, spironolactone, torsemide, triamterene) presented a reduction in AD risk [95]. In another cohort study that followed individuals of at least 65 years of age for six years, it was observed that the use ACEIs or ARBs presented a more protective effect against the onset of AD in males in comparison of other anti-hypertensive drugs. Moreover, the use of ARBs was more effective in preventing AD than ACEIs in both genders [96].

In relation to statins, there is evidence that these drugs can exhibit anti-inflammatory, anti-oxidative, and anti-atherosclerotic effects, especially atorvastatin [97]. It was demonstrated that this drug may improve left ventricular remodeling, protecting ventricular diastolic function in HF [98]. Furthermore, the use of pitavastatin was associated with a lower onset of AF in elderly hypertensive patients [99]. In this context, in animal models, the treatment with simvastatin exhibited a reduced vulnerability to the development of AF [100]. In clinical studies, the previous use of different types of statins, such as atorvastatin, simvastatin, and lovastatin, reduced the incidence of postoperative AF and circulating inflammatory markers [101,102]. However, atorvastatin and pravastatin, as well as other statins, were not effective in preventing arrhythmia, nor in recurrences after pharmacological cardioversion and catheter ablation [103]. With regard to the use of statins in AD, in a cohort study that followed older subjects for eleven years, it was observed that synthetic and hydrophilic statins, such as rosuvastatin, but not fungus-derived and lipophilic statins, such as simvastatin, were associated with a decreased incidence of AD [104]. In a meta-analysis, however, the use of hydrophilic statins was associated with a decreased risk of all-caused dementia (RR 0.877, 95% CI 0.818–0.940, *p* = 0.000) and a possible lower risk of AD (RR 0.619, 95% CI 0.383–1.000, *p* = 0.050), while lipophilic statins showed an association with reduced risk for AD (RR 0.639, 95% CI 0.449–0.908, *p* = 0.013) but not to all caused-dementia (RR 0.738, 95% CI 0.475–1.146, *p* = 0.176) [105].

In relation to colchicine, it appeared to reduce the incidence of postoperative AF, an effect accompanied by plasma reductions in inflammatory mediators such as CRP and IL-6 [106]. However, in another study, the use of this drug did not prevent the development of arrhythmia after surgery [107]. Regarding the use of colchicine in AD, although it was proposed almost two decades ago [108], some years later it was observed that colchicine injections cause cognition impairment and a reduction in acetylcholinesterase levels in the brains of rats, suggesting that the injection of this drug could be used as an animal model for AD [109].

Finally, in relation to the PUFAs, there are studies demonstrating that the clinical use of omega-3 PUFAs presented a role in the primary [110] and secondary (recurrences) prevention of AF [111]. However, these effects were not observed in other trials [112]. Furthermore, its reduction in the incidence of postoperative AF has not been demonstrated in patients who have undergone cardiac surgery [113]. In relation to this approach in AD, the results are also inconclusive. In an experimental study, it was observed lower levels of βAP in the hippocampus of transgenic mice model of AD that were fed with peroxidation-resistant polyunsaturated fatty acids [114]. However, in a clinical interventional study, the dietary supplementation of AD patients with omega-3 PUFAs for four months showed no effects on cognition and mood [115].

The metabolites of other PUFAs, such as epoxy-eicosa-trienoic acids (EETs), which are derivatives of omega-6 arachidonic acid, have also demonstrated anti-inflammatory properties. EETs, as well as other PUFAs metabolites, are converted into diols by the soluble epoxide hydrolase (sEH) enzyme, however the EETs diols formed are less active. Thus, inhibitors of sEH (sEHI) have become a novel therapeutic approach for inflammatory conditions as well as cardio-vascular and neuro-degenerative diseases [116]. In the context of AF, an experimental study evaluated the role of trifluoro-methoxy-phenyl-3-(1-propionyl-piperidine-4-yl)-urea (TPPU), an sEHI, in a pressure-overload-induced atrial remodeling model in murines. These authors observed that animals treated with TPPU presented a reduction in inflammation, atrial fibrosis, and electrical remodeling in atrial myocytes, suggesting sEH inhibition as a therapeutic target for the treatment of AF [117]. In a murine model of myocardial infarction (MI), treatment with two sEHIs, 1-adamantan-1-yl-3-{5-[2-(2-ethoxy-ethoxy)-ethoxy]-pentyl}-urea (AEPU) and *trans*-4-[4-(3-adamantan-1-yl-ureido)-cyclohexyloxy]-benzoic acid (*t*-AUCB), was also evaluated. The inhibition of sEH showed to be effective in the reduction of the infarct size and in the prevention of cardiac electrical remodeling after MI, thus reducing the development of cardiac arrhythmias [118,119]. On the other hand, in a clinical study that evaluated 218 patients with AF who underwent catheter ablation and 268 controls, a polymorphism in the *EPHX2* gene, which encodes human sEH, was associated with a higher risk of AF recurrence after catheter ablation within 12 months (OR 3.2, 95% CI 1.237–8.276, *p* = 0.016) and 24 months (OR 6.076, 95% CI 2.244–16.451, *p* < 0.0001). In this case, it was suggested that this polymorphism may lead to a deficient metabolism of EETs, whose can suppress scar formation after catheter ablation, favoring the recurrence of AF [120].

In relation to AD and EETs, it was shown that βAP reduces EETs synthesis in hippocampal astrocytes and neurons in rat brains [121]. In addition, in the cell culture of the hippocampal astrocytes of neonatal rats pretreated with regioisomers of EETs, an improvement in mitochondrial function and cellular respiration was observed in the presence of βAP. Thus, it was suggested that one of the mechanisms of the toxicity of βAP is the reduction in endogenous EETs, and a cell treatment with exogenous EET was able to reverse this effect [122]. Corroborating this data, increased levels of sEH in the brains of AD transgenic mice were observed, predominantly in hippocampal astrocytes. Moreover, the genetic depletion of sEH in this animal model increased the production of astrocyte-derived anti-inflammatory cytokines and delayed the progression of AD, which was observed by the improvement in behavioral tests and attenuation in βAP plaque deposition. Thus, sEH was suggested as a potential therapeutic target for AD [123]. In line with this, two different models of transgenic mice for AD were treated with three distinct sEHI: TPPU, AS-2586114, and UB-EV-52. It was observed that these three inhibitors reduced the gene expression and the levels of pro-inflammatory cytokines in the brain. Furthermore, they also promoted a reduction in tau hyperphosphorylation and in the number of βAP plaques stained, as well as showing an improvement in cognitive decline, as observed in the behavioral test. In view of this, sEH inhibition was proposed as a potential strategy for AD treatment [124].

### 4.2. Therapies for AF and Its Potential Role in Prevention of Dementia and AD

The treatment with oral anticoagulants (OACs), such as the vitamin K inhibitor warfarin and the novel oral anticoagulants (NOACs) rivaroxaban, apixaban, dabigatran and others, is recommended to patients who presented non-valvular AF and are at high risk to develop thromboembolic events. In this context, in a study involving 2605 patients with AF treated with warfarin who were followed for a median period of four years, it was observed that a rigorous and consistent anticoagulation therapy was related to a decreased risk of developing dementia in general [125]. Moreover, in another study that followed 5254 patients with AF from June 2010 to December 2014, in treatment with warfarin or NOACs, it was found that those who were taking the non-vitamin K inhibitors presented a 43% lower incidence of strokes, transient ischemic attacks, and dementia (HR 0.57, 95% CI 0.17–1.97, *p* = 0.38) in comparison to those using warfarin [126].

As regards rhythm management in patients with AF, a study compared 4212 patients who underwent AF ablation to 16,848 patients with AF but no ablation and 16,848 with no AF. It was observed that in those who underwent ablation therapy, the incidence of AD was 0.2%, while in those that did not receive this therapy it was 0.9%, and in those without AF it was 0.5%. In this way, ablation therapy in AF patients was associated with a reduced AD risk [127].

### 4.3. Paradigm in the Treatment of Patients With AD Who Have AF

Currently, the treatment approved for mild to moderate AD symptoms includes cholinesterase inhibitor (ChEI) drugs such as donepezil, rivastigmine, and galantamine. However, these drugs present some side effects, such as increased blood pressure and decreased pulse. The occurrence of syncope in patients who used donepezil, due to cardiovascular abnormalities, has also been reported [128]. Due to this, the use of ChEI is not recommended for AD patients who have cardiovascular comorbidities, including supraventricular conduction problems such as atrial fibrillation and a flutter. However, there is a lack of guidelines and standardization for prescription of this class of drugs in these conditions [129]. Moreover, in a cohort study, an association was reported between the use of ChEI in patients with dementia and a reduced risk of ischemic strokes (HR 0.508, 95% CI 0.434–0.594, *p* < 0.001). Considering that strokes are also a mainly complication for AF patients and that subjects whose present both AD and AF could benefit from this effect of using ChEI, more evidence is needed to maintain the non-recommendation in the use of these drugs for patients with AD who have also arrhythmia [130].

## 5. Final Considerations

**Figure 1:**
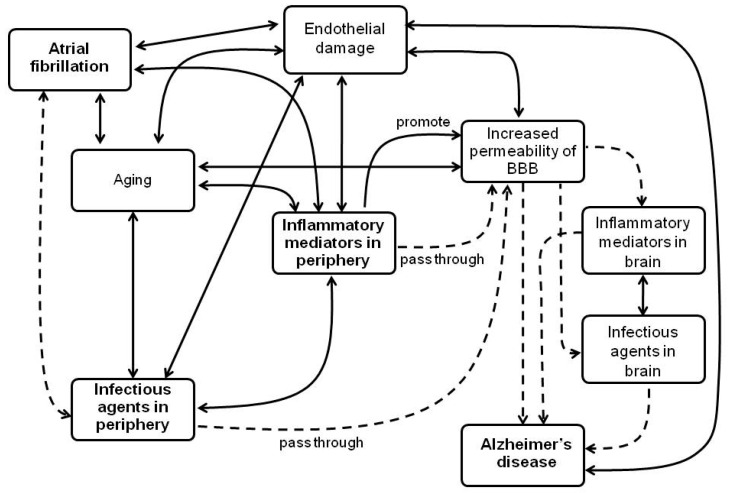
Proposed mechanisms that can correlate AF and AD by inflammatory and infectious processes. AF can cause endothelial damage due to the turbulent and static flow within the atrium. Thus, it can initiate an inflammatory response and thrombogensis, becoming a chronic situation. When AF is persistent, it also may promote a decrease in cardiac output, leading to a chronic cerebral hypoperfusion and hypoxia. These factors can lead to endothelial damage, increasing the release of inflammatory mediators and altering the permeability of the blood–brain barrier (BBB). Peripheral inflammatory molecules can pass through the BBB and promote neuroinflammation that can lead to AD. Individuals with AF can also be more susceptible to infection by some agents. Thus, the inflammatory state in these patients could become exacerbated, favoring the alteration in the permeability of the BBB. In this way, these infectious agents could pass to the brain and may have some effect on the pathophysiology of AD, in association with β-amyloid peptide (βAP) plaques deposition. It is worth mentioning that aging is associated with both AF and AD, as well as with a chronic inflammatory state, endothelial damage, the increased permeability of the BBB and a higher risk of acquiring infections due to a disbalance in the immune system. Abbreviation: BBB, blood brain barrier.

In view of the above, it was possible to observe a series of evidence correlating AF and AD, suggesting inflammatory and infectious processes as potential links between both diseases. AF is not considered a risk factor for AD, however, besides an increase in the BBB permeability that occurs with age, it can also be promoted by other factors related to AF, such as hypoperfusion and hypoxia in the brain and the release of inflammatory mediators in the blood. Thus, it may be related to the pathogenesis of AD, regardless of the occurrence of strokes. In addition, some infectious agents that may be associated to AF can also play a role in AD pathology, suggesting a possible link between these conditions. In Figure 1, there is a graphical abstract summarizing the proposed mechanisms involved in these complex processes. Moreover, in relation to pharmacological approaches, although anti-inflammatory strategies have not exhibited to date great results in the management of patients with AF, these drugs have shown potential effects in the prevention of developing AD in the general population. In addition, current studies have shown the potential benefits of the use of anticoagulants in patients with AF in the prevention of dementia and AD. In this context, more long-scale and prospective studies should be performed to evaluate this beneficial effect. In general, it is possible that further knowledge regarding the inflammatory and infectious aspects relating to AF and AD is required and may provide opportunities for innovation in the therapy and management of patients with these clinical situations.

## Figures and Tables

**Table 1:** Main inflammatory molecules related to both atrial fibrillation (AF) and Alzheimer’s disease (AD).

Parameter	Atrial Fibrillation	Alzheimer’s Disease
IL-1β	In the atrium of patients with AF, in comparison to controls, an increase in pro-inflammatory macrophages was observed, which was associated with a higher secretion of IL-1β [37]. It was also related to an up-regulation of IL-1β mRNA in the atrium of patients with persistent AF in comparison to those with paroxysmal AF and the control group [43].	IL-1β demonstrated a key function in the deposition of βAP plaques [44]. Higher levels of IL-1β were found in the serum samples of both patients with AD and mild cognition impairment (MCI), leading to the consideration that this cytokine is produced in the beginning of the disease and remains elevated after the establishment of AD [23,45].
IL-6	IL-6 was related to the development of AF after coronary artery bypass grafting (CABG) [38]. IL-6 was also associated with the recurrence of AF after ablation therapy [46].	IL-6 was associated with βAP plaques in the hippocampus and cortex in the AD brain [47] and to abnormally hyperphosphorylated tau protein [48]. Higher levels of IL-6 were found in the serum of AD patients compared to those with MCI and the control group [40].
TNF	Higher levels of TNF were detected in patients with persistent and permanent AF in comparison to those with paroxysmal AF [39]. The TNF values found in the plasma of patients with chronic non-valvular AF were also considered as predictors for the development of ischemic strokes [49].	TNF was associated with an increase in βAP production in an experimental study [41]. In a meta-analysis, an up-regulation of TNF in the blood and CSF samples of patients with AD was reported, especially in those in severe stages of the disease [50].
IL-10	Increased levels of IL-10 were found in peripheral blood samples of AF patients compared to the controls, and higher concentrations were detected in patients with persistent and permanent AF compared to those with paroxysmal AF [39]. Higher serum levels of IL-10 were also associated with the development of AF after CABG [51].	IL-10 was related to the accumulation of βAP in an animal model [52]. In a clinical study, AD patients exhibited higher serum levels of IL-10 than the controls, suggesting that peripheral levels of IL-10 may be related to AD pathogenesis [42].
MCP-1	Higher levels of monocyte chemo-attractant protein (MCP)-1 were found in venous blood samples from patients with AF compared to the controls [39]. Elevated levels of this marker were also found in the plasma of patients with paroxysmal and permanent AF in relation to individuals with sinus rhythm [53].	Higher levels of MCP-1 were found in AD patients compared with MCI patients and controls, and the highest levels were assessed in severe AD patients. Thus, higher plasmatic levels of MCP-1 were associated with a greater severity of AD [54].

Abbreviations: AD: Alzheimer’s disease; βAP: β-amyloid peptide; CABG: coronary artery bypass graft; IL: interleukin; MCI: mild cognitive impairment; TNF: tumor necrosis factor; CSF: cerebrospinal fluid; MCP: monocyte chemo-attractant protein.

## Abbreviations

ACEIs	Angiotensin converting enzyme inhibitors
AD	Alzheimer’s disease
AEPU	1-adamantan-1-yl-3-{5-[2-(2-ethoxy-ethoxy)-ethoxy]-pentyl}-urea
AF	Atrial fibrillation
ARBs	Angiotensin-1 receptor blockers
BBB	Blood brain barrier
BCSFB	Blood-cerebrospinal fluid barrier
CAIDE	Cardiovascular Risk Factors, Aging and Dementia
ChEI	Cholinesterase inhibitors
CI	Confidence interval
CNS	Central nervous system
COX	Cyclooxygenase
CRP	C reactive protein
CSF	Cerebrospinal fluid
EETs	Epoxy-eicosa-trienoic acids
*EPHX2*	Epoxide hydrolase 2
HF	Heart failure
HR	Hazard ratio
HSPs	Heat shock proteins
HSV	Herpes simplex virus
Ig	Immunoglobulin
IL	Interleukin
MCI	Mild cognition impairment
MCP	Monocyte chemo-attractant protein
MI	Myocardial infarction
mRNA	Messenger ribonucleic acid
NOACs	Novel oral anticoagulants
NSAIDs	Non-steroidal anti-inflammatory drugs
OACs	Oral anticoagulants
OR	Odds ratio
PUFAs	Poly-unsaturated fatty acids
RAAS	Renin-angiotensin-aldosterone system
RR	Relative risk
sEH	Soluble epoxide hydrolase
sEHI	Soluble epoxide hydrolase inhibitors
*t*-AUCB	*trans*-4-[4-(3-adamantan-1-yl-ureido)-cyclohexyloxy]-benzoic acid
TGF	Transforming growth factor
TNF	Tumor necrosis factor
TPPU	Trifluoro-methoxy-phenyl-3-(1-propionyl-piperidine-4-yl)-urea
βAP	β-amyloid peptide

## References

[1] Savelieva I., Kakouros N., Kourliouros A., Camm A.J. (2011). Upstream therapies for management of atrial fibrillation: Review of clinical evidence and implications for European Society of Cardiology guidelines. Part I: Primary prevention. Europace.

[2] Vlachos K., Letsas K.P., Korantzopoulos P., Liu T., Georgopoulos S., Bakalakos A., Karamichalakis N., Xydonas S., Efremidis M., Sideris A. (2016). Prediction of atrial fibrillation development and progression: Current perspectives. World J. Cardiol..

[3] Ihara M., Washida K. (2018). Linking atrial fibrillation with alzheimer’s disease: Epidemiological, pathological, and mechanistic evidence. J. Alzheimers Dis..

[4] Dietzel J., Haeusler K.G., Endres M. (2017). Does atrial fibrillation cause cognitive decline and dementia?. EP Eur..

[5] Sposato L.A., Vargas E.R., Riccio P.M., Toledo J.B., Trojanowski J.Q., Kukull W.A., Cipriano L.E., Nucera A., Whitehead S.N., Hachinski V. (2017). Milder Alzheimer’s disease pathology in heart failure and atrial fibrillation. Alzheimers Dement..

[6] Chen L.Y., Norby F.L., Gottesman R.F., Mosley T.H., Soliman E.Z., Agarwal S.K., Loehr L.R., Folsom A.R., Coresh J., Alonso A. (2018). Association of Atrial Fibrillation with Cognitive Decline and Dementia Over 20 Years: The ARIC-NCS (Atherosclerosis Risk in Communities Neurocognitive Study). J. Am. Heart Assoc..

[7] Qiu C., Winblad B., Marengoni A., Klarin I., Fastbom J., Fratiglioni L. (2006). Heart failure and risk of dementia and Alzheimer disease: A population-based cohort study. Arch. Intern. Med..

[8] Dublin S., Anderson M.L., Haneuse S.J., Heckbert S.R., Crane P.K., Breitner J.C., McCormick W., Bowen J.D., Teri L., McCurry S.M. (2011). Atrial fibrillation and risk of dementia: A prospective cohort study. J. Am. Geriatr. Soc..

[9] Rusanen M., Kivipelto M., Levälahti E., Laatikainen T., Tuomilehto J., Soininen H., Ngandu T. (2014). Heart diseases and long-term risk of dementia and Alzheimer’s disease: A population-based CAIDE study. J. Alzheimers Dis..

[10] Di Nisio M., Prisciandaro M., Rutjes A.W., Russi I., Maiorini L., Porreca E. (2015). Dementia in patients with atrial fibrillation and the value of the H achinski ischemic score. Geriatr. Gerontol. Int..

[11] Bunch T.J., Weiss J.P., Crandall B.G., May H.T., Bair T.L., Osborn J.S., Anderson J.L., Muhlestein J.B., Horne B.D., Lappe D.L. (2010). Atrial fibrillation is independently associated with senile, vascular, and Alzheimer’s dementia. Heart Rhythm.

[12] Knecht S., Oelschläger C., Duning T., Lohmann H., Albers J., Stehling C., Heindel W., Breithardt G., Berger K., Ringelstein E.B. (2008). Atrial fibrillation in stroke-free patients is associated with memory impairment and hippocampal atrophy. Eur. Heart J..

[13] Dublin S., Anderson M.L., Heckbert S.R., Hubbard R.A., Sonnen J.A., Crane P.K., Montine T.J., Larson E.B. (2013). Neuropathologic changes associated with atrial fibrillation in a population-based autopsy cohort. J. Gerontol. A Biol. Sci. Med. Sci..

[14] DeSimone C.V., Graff-Radford J., El-Harasis M.A., Rabinstein A.A., Asirvatham S.J., Holmes D.R. (2017). Cerebral amyloid angiopathy: Diagnosis, clinical implications, and management strategies in atrial fibrillation. J. Am. Coll. Cardiol..

[15] Ding M., Qiu C. (2018). Atrial Fibrillation, Cognitive Decline, and Dementia: An Epidemiologic Review. Curr. Epidemiol. Rep..

[16] Shah A.D., Merchant F.M., Delurgio D.B. (2016). Atrial fibrillation and risk of dementia/cognitive decline. J. Atr. Fibrillation.

[17] Jefferson A.L., Beiser A.S., Himali J.J., Seshadri S., O’Donnell C.J., Manning W.J., Wolf P.A., Au R., Benjamin E.J. (2015). Low cardiac index is associated with incident dementia and Alzheimer disease: The Framingham Heart Study. Circulation.

[18] Liu F., Grundke-Iqbal I., Iqbal K., Gong C.X. (2005). Contributions of protein phosphatases PP1, PP2A, PP2B and PP5 to the regulation of tau phosphorylation. Eur. J. Neurosci..

[19] Troncone L., Luciani M., Coggins M., Wilker E.H., Ho C.-Y., Codispoti K.E., Frosch M.P., Kayed R., Del Monte F. (2016). Aβ amyloid pathology affects the hearts of patients with Alzheimer’s disease: Mind the Heart. J. Am. Coll. Cardiol..

[20] Harada M., Van Wagoner D.R., Nattel S. (2015). Role of inflammation in atrial fibrillation pathophysiology and management. Circ. J..

[21] Korantzopoulos P., Letsas K.P., Tse G., Fragakis N., Goudis C.A., Liu T. (2018). Inflammation and atrial fibrillation: A comprehensive review. J. Arrhythm..

[22] Wyss-Coray T., Rogers J. (2012). Inflammation in Alzheimer disease—A brief review of the basic science and clinical literature. Cold Spring Harb. Perspect. Med..

[23] Kinney J.W., Bemiller S.M., Murtishaw A.S., Leisgang A.M., Lamb B.T. (2018). Inflammation as a central mechanism in Alzheimer’s disease. Alzheimers Dement..

[24] Heppner F.L., Ransohoff R.M., Becher B. (2015). Immune attack: The role of inflammation in Alzheimer disease. Nat. Rev. Neurosci..

[25] Andrew P., Montenero A.S. (2007). Is there a link between atrial fibrillation and certain bacterial infections?. J. Cardiovasc. Med. (Hagerstown).

[26] Sochocka M., Zwolinska K., Leszek J. (2017). The infectious etiology of Alzheimer’s disease. Curr. Neuropharmacol..

[27] Magalhães L.P., Figueiredo M.J.O., Cintra F.D., Saad E.B., Kuniyishi R.R., Teixeira R.A. (2016). II Diretrizes Brasileiras de Fibrilação Atrial. Arq. Bras. Cardiol..

[28] Hu Y.F., Chen Y.J., Lin Y.J., Chen S.A. (2015). Inflammation and the pathogenesis of atrial fibrillation. Nat. Rev. Cardiol..

[29] Watson T., Shantsila E., Lip G.Y. (2009). Mechanisms of thrombogenesis in atrial fibrillation: Virchow’s triad revisited. Lancet.

[30] Ott B.R., Jones R., Daiello L.A., Monte S.d.L., Stopa E.G., Johanson C.E., Denby C., Grammas P. (2018). Blood-Cerebrospinal Fluid Barrier Gradients in Mild Cognitive Impairment and Alzheimer’s Disease: Relationship to Inflammatory Cytokines and Chemokines. Front. Aging Neurosci..

[31] Farrall A.J., Wardlaw J.M. (2009). Blood–brain barrier: Ageing and microvascular disease–systematic review and meta-analysis. Neurobiol. Aging.

[32] Johanson C., Stopa E., Daiello L., De la Monte S., Keane M., Ott B. (2018). Disrupted blood-CSF barrier to urea and creatinine in mild cognitive impairment and Alzheimer’s disease. J. Alzheimers Dis. Parkinsonism.

[33] Chung H.Y., Kim D.H., Lee E.K., Chung K.W., Chung S., Lee B., Seo A.Y., Chung J.H., Jung Y.S., Im E. (2019). Redefining Chronic Inflammation in Aging and Age-Related Diseases: Proposal of the Senoinflammation Concept. Aging Dis..

[34] Elwood E., Lim Z., Naveed H., Galea I. (2017). The effect of systemic inflammation on human brain barrier function. Brain. Behav. Immun..

[35] Di Marco L.Y., Venneri A., Farkas E., Evans P.C., Marzo A., Frangi A.F. (2015). Vascular dysfunction in the pathogenesis of Alzheimer’s disease—A review of endothelium-mediated mechanisms and ensuing vicious circles. Neurobiol. Dis..

[36] Takeda S., Sato N., Morishita R. (2014). Systemic inflammation, blood-brain barrier vulnerability and cognitive/non-cognitive symptoms in Alzheimer disease: Relevance to pathogenesis and therapy. Front. Aging Neurosci..

[37] Sun Z., Zhou D., Xie X., Wang S., Wang Z., Zhao W., Xu H., Zheng L. (2016). Cross-talk between macrophages and atrial myocytes in atrial fibrillation. Basic Res. Cardiol..

[38] Kaireviciute D., Blann A.D., Balakrishnan B., Lane D.A., Patel J.V., Uzdavinys G., Norkunas G., Kalinauskas G., Sirvydis V., Aidietis A. (2010). Characterisation and validity of inflammatory biomarkers in the prediction of post-operative atrial fibrillation in coronary artery disease patients. Thromb. Haemost..

[39] Li J., Solus J., Chen Q., Rho Y.H., Milne G., Stein C.M., Darbar D. (2010). Role of inflammation and oxidative stress in atrial fibrillation. Heart Rhythm.

[40] Kim Y.S., Lee K.J., Kim H. (2017). Serum tumour necrosis factor-α and interleukin-6 levels in Alzheimer’s disease and mild cognitive impairment. Psychogeriatrics.

[41] Paouri E., Tzara O., Zenelak S., Georgopoulos S. (2017). Genetic Deletion of Tumor Necrosis Factor-α Attenuates Amyloid-β Production and Decreases Amyloid Plaque Formation and Glial Response in the 5XFAD Model of Alzheimer’s Disease. J. Alzheimers Dis..

[42] D’Anna L., Abu-Rumeileh S., Fabris M., Pistis C., Baldi A., Sanvilli N., Curcio F., Gigli G.L., D’Anna S., Valente M. (2017). Serum interleukin-10 levels correlate with cerebrospinal fluid amyloid beta deposition in Alzheimer disease patients. Neurodegener Dis..

[43] Wu W., Ke D., Xu C., Deng Y., Chen L., Zhang J., Lin Y., Hu X. (2006). Collagen type I and Interleukin-1 beta gene expression in human atria during atrial fibrillation. Zhonghua Nei Ke Za Zhi.

[44] Akiyama H., Barger S., Barnum S., Bradt B., Bauer J., Cole G.M., Cooper N.R., Eikelenboom P., Emmerling M., Fiebich B.L. (2000). Inflammation and Alzheimer’s disease. Neurobiol. Aging.

[45] Forlenza O.V., Diniz B.S., Talib L.L., Mendonça V.A., Ojopi E.B., Gattaz W.F., Teixeira A.L. (2009). Increased serum IL-1β level in Alzheimer’s disease and mild cognitive impairment. Dement. Geriatr. Cogn. Disord..

[46] Henningsen K.M., Therkelsen S.K., Bruunsgaard H., Krabbe K.S., Pedersen B.K., Svendsen J.H. (2009). Prognostic impact of hs-CRP and IL-6 in patients with persistent atrial fibrillation treated with electrical cardioversion. Scand. J. Clin. Lab. Investig..

[47] Hampel H., Haslinger A., Scheloske M., Padberg F., Fischer P., Unger J., Teipel S.J., Neumann M., Rosenberg C., Oshida R. (2005). Pattern of interleukin-6 receptor complex immunoreactivity between cortical regions of rapid autopsy normal and Alzheimer’s disease brain. Eur. Arch. Psychiatry Clin. Neurosci..

[48] Quintanilla R.A., Orellana D.I., González-Billault C., Maccioni R.B. (2004). Interleukin-6 induces Alzheimer-type phosphorylation of tau protein by deregulating the cdk5/p35 pathway. Exp. Cell Res..

[49] Pinto A., Tuttolomondo A., Casuccio A., Di Raimondo D., Di Sciacca R., Arnao V., Licata G. (2009). Immuno-inflammatory predictors of stroke at follow-up in patients with chronic non-valvular atrial fibrillation (NVAF). Clin. Sci..

[50] Brosseron F., Krauthausen M., Kummer M., Heneka M.T. (2014). Body fluid cytokine levels in mild cognitive impairment and Alzheimer’s disease: A comparative overview. Mol. Neurobiol..

[51] Hak L., Mysliwska J., Wieckiewicz J., Szyndler K., Siebert J., Rogowski J. (2009). Interleukin-2 as a predictor of early postoperative atrial fibrillation after cardiopulmonary bypass graft (CABG). J. Interferon Cytokine Res..

[52] Guillot-Sestier M.-V., Doty K.R., Gate D., Rodriguez J., Leung B.P., Rezai-Zadeh K., Town T. (2015). Il10 deficiency rebalances innate immunity to mitigate Alzheimer-like pathology. Neuron.

[53] Alegret J.M., Aragonès G., Elosua R., Beltrán-Debón R., Hernández-Aguilera A., Romero-Menor C., Camps J., Joven J. (2013). The relevance of the association between inflammation and atrial fibrillation. Eur. J. Clin. Investig..

[54] Lee W.-J., Liao Y.-C., Wang Y.-F., Lin I.-F., Wang S.-J., Fuh J.-L. (2018). Plasma MCP-1 and cognitive decline in patients with Alzheimer’s disease and mild cognitive impairment: A two-year follow-up study. Sci. Rep..

[55] Guazzi M., Arena R. (2009). Endothelial dysfunction and pathophysiological correlates in atrial fibrillation. Heart.

[56] Bunch T.J., Galenko O., Graves K.G., Jacobs V., May H.T. (2019). Atrial Fibrillation and Dementia: Exploring the Association, Defining Risks and Improving Outcomes. Arrhythm. Electrophysiol. Rev..

[57] Guo Y., Lip G.Y., Apostolakis S. (2012). Inflammation in atrial fibrillation. J. Am. Coll. Cardiol..

[58] Siasos G., Mazaris S., Zisimos K., Oikonomou E., Kokkou E., Konsola T., Mourouzis K., Vavuranakis M., Zografos T., Zaromitidou M. (2015). The impact of atrial fibrillation on endothelial dysfunction. J. Am. Coll. Cardiol..

[59] Weymann A., Sabashnikov A., Ali-Hasan-Al-Saegh S., Popov A.-F., Mirhosseini S.J., Baker W.L., Lotfaliani M., Liu T., Dehghan H., Yavuz S. (2017). Predictive role of coagulation, fibrinolytic, and endothelial markers in patients with atrial fibrillation, stroke, and thromboembolism: A meta-analysis, meta-regression, and systematic review. Med. Sci. Monit. Basic Res..

[60] Kelleher R.J., Soiza R.L. (2013). Evidence of endothelial dysfunction in the development of Alzheimer’s disease: Is Alzheimer’sa vascular disorder?. Am. J. Cardiovasc. Dis..

[61] Herrera M.D., Mingorance C., Rodríguez-Rodríguez R., De Sotomayor M.A. (2010). Endothelial dysfunction and aging: An update. Ageing Res. Rev..

[62] Montenero A.S., Mollichelli N., Zumbo F., Antonelli A., Dolci A., Barberis M., Sirolla C., Staine T., Fiocca L., Bruno N. (2005). Helicobacter pylori and atrial fibrillation: A possible pathogenic link. Heart.

[63] Kountouras J., Boziki M., Gavalas E., Zavos C., Deretzi G., Grigoriadis N., Tsolaki M., Chatzopoulos D., Katsinelos P., Tzilves D. (2009). Increased cerebrospinal fluid Helicobacter pylori antibody in Alzheimer’s disease. Int. J. Neurosci..

[64] Huang W.-S., Yang T.-Y., Shen W.-C., Lin C.-L., Lin M.-C., Kao C.-H. (2014). Association between Helicobacter pylori infection and dementia. J. Clin. Neurosci..

[65] Beydoun M.A., Beydoun H.A., Shroff M.R., Kitner-Triolo M.H., Zonderman A.B. (2013). Helicobacter pylori seropositivity and cognitive performance among US adults: Evidence from a large national survey. Psychosom. Med..

[66] Gravina A.G., Zagari R.M., De Musis C., Romano L., Loguercio C., Romano M. (2018). Helicobacter pylori and extragastric diseases: A review. World J. Gastroenterol..

[67] Chiang C.-H., Huang C.-C., Chan W.-L., Huang P.-H., Chen Y.-C., Chen T.-J., Lin S.-J., Chen J.-W., Leu H.-B. (2013). Herpes simplex virus infection and risk of atrial fibrillation: A nationwide study. Int. J. Cardiol..

[68] Lövheim H., Gilthorpe J., Johansson A., Eriksson S., Hallmans G., Elgh F. (2015). Herpes simplex infection and the risk of Alzheimer’s disease: A nested case-control study. Alzheimers Dement..

[69] Lövheim H., Gilthorpe J., Adolfsson R., Nilsson L.-G., Elgh F. (2015). Reactivated herpes simplex infection increases the risk of Alzheimer’s disease. Alzheimers Dement..

[70] Wozniak M., Mee A., Itzhaki R. (2009). Herpes simplex virus type 1 DNA is located within Alzheimer’s disease amyloid plaques. J. Pathol..

[71] Itzhaki R.F. (2018). Corroboration of a major role for herpes simplex virus type 1 in Alzheimer’s disease. Front Aging Neurosci..

[72] Krijthe B.P., Heeringa J., Hofman A., Franco O.H., Stricker B.H. (2014). Non-steroidal anti-inflammatory drugs and the risk of atrial fibrillation: A population-based follow-up study. BMJ Open.

[73] Schmidt M., Christiansen C.F., Mehnert F., Rothman K.J., Sørensen H.T. (2011). Non-steroidal anti-inflammatory drug use and risk of atrial fibrillation or flutter: Population based case-control study. BMJ.

[74] Aslam A.K., Vasavada B.C., Sacchi T.J., Khan I.A. (2001). Atrial fibrillation associated with systemic lupus erythematosus and use of methylprednisolone. Am. J. Ther..

[75] Whelton A. (2001). Renal aspects of treatment with conventional nonsteroidal anti-inflammatory drugs versus cyclooxygenase-2–specific inhibitors. Am. J. Med..

[76] Zhang C., Wang Y., Wang D., Zhang J., Zhang F. (2018). NSAID Exposure and Risk of Alzheimer’s Disease: An Updated Meta-Analysis from Cohort Studies. Front. Aging Neurosci..

[77] Li X., Kaida-Yip F., Zabel M. (2018). NSAID Use and the Prevention of Alzheimer’s Disease: A Meta-Analysis (P6. 184). Neurology.

[78] Shiroshita-Takeshita A., Brundel B.J., Lavoie J., Nattel S. (2006). Prednisone prevents atrial fibrillation promotion by atrial tachycardia remodeling in dogs. Cardiovasc. Res..

[79] Ho K.M., Tan J.A. (2009). Benefits and risks of corticosteroid prophylaxis in adult cardiac surgery: A dose-response meta-analysis. Circulation.

[80] Liu C., Wang J., Yiu D., Liu K. (2014). The Efficacy of Glucocorticoids for the Prevention of Atrial Fibrillation, or Length of Intensive Care Unite or Hospital Stay After Cardiac Surgery: A Meta-Analysis. Cardiovasc. Ther..

[81] Koyama T., Tada H., Sekiguchi Y., Arimoto T., Yamasaki H., Kuroki K., Machino T., Tajiri K., Zhu X.D., Kanemoto-Igarashi M. (2010). Prevention of atrial fibrillation recurrence with corticosteroids after radiofrequency catheter ablation: A randomized controlled trial. J. Am. Coll. Cardiol..

[82] Christiansen C.F., Christensen S., Mehnert F., Cummings S.R., Chapurlat R.D., Sørensen H.T. (2009). Glucocorticoid use and risk of atrial fibrillation or flutter: A population-based, case-control study. Arch. Intern. Med..

[83] Van der Hooft C.S., Heeringa J., Brusselle G.G., Hofman A., Witteman J.C., Kingma J.H., Sturkenboom M.C., Stricker B.H.C. (2006). Corticosteroids and the risk of atrial fibrillation. Arch. Intern. Med..

[84] Wei L., MacDonald T.M., Walker B.R. (2004). Taking glucocorticoids by prescription is associated with subsequent cardiovascular disease. Ann. Intern. Med..

[85] Beeri M.S., Schmeidler J., Lesser G.T., Maroukian M., West R., Leung S., Wysocki M., Perl D.P., Purohit D.P., Haroutunian V. (2012). Corticosteroids, but not NSAIDs, are associated with less Alzheimer neuropathology. Neurobiol. Aging.

[86] Aisen P.S., Davis K., Berg J., Schafer K., Campbell K., Thomas R., Weiner M., Farlow M., Sano M., Grundman M. (2000). A randomized controlled trial of prednisone in Alzheimer’s disease. Neurology.

[87] Libro R., Bramanti P., Mazzon E. (2017). Endogenous glucocorticoids: Role in the etiopathogenesis of Alzheimer’s disease. Neuro Endocrinol. Lett..

[88] Mandal K., Torsney E., Poloniecki J., Camm A.J., Xu Q., Jahangiri M. (2005). Association of High Intracellular, But Not Serum, Heat Shock Protein 70 With Postoperative Atrial Fibrillation. Ann. Thorac. Cardiovasc. Surg..

[89] Hu Y.-F., Yeh H.-I., Tsao H.-M., Tai C.-T., Lin Y.-J., Chang S.-L., Lo L.-W., Tuan T.-C., Suenari K., Li C.-H. (2012). Electrophysiological correlation and prognostic impact of heat shock protein 27 in atrial fibrillation. Circ. Arrhythm. Electrophysiol..

[90] Shi J.-Q., Shen W., Chen J., Wang B.-R., Zhong L.-L., Zhu Y.-W., Zhu H.-Q., Zhang Q.-Q., Zhang Y.-D., Xu J. (2011). Anti-TNF-α reduces amyloid plaques and tau phosphorylation and induces CD11c-positive dendritic-like cell in the APP/PS1 transgenic mouse brains. Brain Res..

[91] Caccamo A., Majumder S., Richardson A., Strong R., Oddo S. (2010). Molecular interplay between mammalian target of rapamycin (mTOR), amyloid-β, and tau effects on cognitive impairments. J. Biol. Chem..

[92] Tweedie D., Ferguson R.A., Fishman K., Frankola K.A., Van Praag H., Holloway H.W., Luo W., Li Y., Caracciolo L., Russo I. (2012). Tumor necrosis factor-α synthesis inhibitor 3, 6′-dithiothalidomide attenuates markers of inflammation, Alzheimer pathology and behavioral deficits in animal models of neuroinflammation and Alzheimer’s disease. J. Neuroinflammation.

[93] Hsieh Y.-C., Hung C.-Y., Li C.-H., Liao Y.-C., Huang J.-L., Lin C.-H., Wu T.-J. (2016). Angiotensin-receptor blocker, angiotensin-converting enzyme inhibitor, and risks of atrial fibrillation: A nationwide cohort study. Medicine.

[94] Zhang Q., Chen Y., Liu Q., Shan Q. (2016). Effects of renin–angiotensin–aldosterone system inhibitors on mortality, hospitalization, and diastolic function in patients with HFpEF. Herz.

[95] Yasar S., Xia J., Yao W., Furberg C.D., Xue Q.-L., Mercado C.I., Fitzpatrick A.L., Fried L.P., Kawas C.H., Sink K.M. (2013). Antihypertensive drugs decrease risk of Alzheimer disease: Ginkgo Evaluation of Memory Study. Neurology.

[96] Barthold D., Joyce G., Wharton W., Kehoe P., Zissimopoulos J. (2018). The association of multiple anti-hypertensive medication classes with Alzheimer’s disease incidence across sex, race, and ethnicity. PLoS ONE.

[97] Feng B., Xu L., Wang H., Yan X., Xue J., Liu F., Hu J.-F. (2011). Atorvastatin exerts its anti-atherosclerotic effects by targeting the receptor for advanced glycation end products. Biochim. Biophys. Acta.

[98] An L., An S., Jia Z., Wang H., Yang Z., Xu C., Teng X., Wang J., Liu X., Cao Q. (2019). Atorvastatin improves left ventricular remodeling and cardiac function in rats with congestive heart failure by inhibiting RhoA/Rho kinase-mediated endothelial nitric oxide synthase. Exp. Ther. Med..

[99] Warita S., Kawasaki M., Tanaka R., Ono K., Kojima T., Hirose T., Iwama M., Watanabe T., Nishigaki K., Takemura G. (2012). Effects of pitavastatin on cardiac structure and function and on prevention of atrial fibrillation in elderly hypertensive patients. Circ. J..

[100] Shiroshita-Takeshita A., Schram G., Lavoie J., Nattel S. (2004). Effect of simvastatin and antioxidant vitamins on atrial fibrillation promotion by atrial-tachycardia remodeling in dogs. Circulation.

[101] Maesen B., Nijs J., Maessen J., Allessie M., Schotten U. (2011). Post-operative atrial fibrillation: A maze of mechanisms. Europace.

[102] Liakopoulos O.J., Choi Y.-H., Kuhn E.W., Wittwer T., Borys M., Madershahian N., Wassmer G., Wahlers T. (2009). Statins for prevention of atrial fibrillation after cardiac surgery: A systematic literature review. J. Thorac. Cardiovasc. Surg..

[103] Bang C.N., Greve A.M., Abdulla J., Køber L., Gislason G.H., Wachtell K. (2013). The preventive effect of statin therapy on new-onset and recurrent atrial fibrillation in patients not undergoing invasive cardiac interventions: A systematic review and meta-analysis. Int. J. Cardiol..

[104] Sinyavskaya L., Gauthier S., Renoux C., Dell’Aniello S., Suissa S., Brassard P. (2018). Comparative effect of statins on the risk of incident Alzheimer disease. Neurology.

[105] Chu C.-S., Tseng P.-T., Stubbs B., Chen T.-Y., Tang C.-H., Li D.-J., Yang W.-C., Chen Y.-W., Wu C.-K., Veronese N. (2018). Use of statins and the risk of dementia and mild cognitive impairment: A systematic review and meta-analysis. Sci. Rep..

[106] Deftereos S., Giannopoulos G., Kossyvakis C., Efremidis M., Panagopoulou V., Kaoukis A., Raisakis K., Bouras G., Angelidis C., Theodorakis A. (2012). Colchicine for prevention of early atrial fibrillation recurrence after pulmonary vein isolation: A randomized controlled study. J. Am. Coll. Cardiol..

[107] Imazio M., Brucato A., Ferrazzi P., Pullara A., Adler Y., Barosi A., Caforio A.L., Cemin R., Chirillo F., Comoglio C. (2014). Colchicine for prevention of postpericardiotomy syndrome and postoperative atrial fibrillation: The COPPS-2 randomized clinical trial. JAMA.

[108] Aisen P.S., Marin D.B., Brickman A.M., Santoro J., Fusco M. (2001). Pilot tolerability studies of hydroxychloroquine and colchicine in Alzheimer disease. Alzheimer Dis. Assoc. Disord..

[109] Kumar A., Seghal N., Naidu P.S., Padi S.S., Goyal R. (2007). Colchicines-induced neurotoxicity as an animal model of sporadic dementia of Alzheimer’s type. Pharmacol. Rep..

[110] Wu J.H., Lemaitre R.N., King I.B., Song X., Sacks F.M., Rimm E.B., Heckbert S.R., Siscovick D.S., Mozaffarian D. (2012). Association of Plasma Phospholipid Long-Chain Omega-3 Fatty Acids with Incident Atrial Fibrillation in Older AdultsClinical Perspective: The Cardiovascular Health Study. Circulation.

[111] Kumar S., Sutherland F., Morton J.B., Lee G., Morgan J., Wong J., Eccleston D.E., Voukelatos J., Garg M.L., Sparks P.B. (2012). Long-term omega-3 polyunsaturated fatty acid supplementation reduces the recurrence of persistent atrial fibrillation after electrical cardioversion. Heart Rhythm.

[112] Nigam A., Talajic M., Roy D., Nattel S., Lambert J., Nozza A., Jones P., Ramprasath V.R., O’Hara G., Kopecky S. (2014). Fish oil for the reduction of atrial fibrillation recurrence, inflammation, and oxidative stress. J. Am. Coll. Cardiol..

[113] Zhang B., Zhen Y., Tao A., Bao Z., Zhang G. (2014). Polyunsaturated fatty acids for the prevention of atrial fibrillation after cardiac surgery: An updated meta-analysis of randomized controlled trials. J. Cardiol..

[114] Shchepinov M., Mattson M., Bennett B. (2017). A new treatment Paradigm for Neurodegeneration: Peroxidation-resistant polyunsaturated fatty acids (D-PUFA) lower brain amyloid beta and oxidation markers, and reverse cognition impairment in vivo (P4. 090). Neurology.

[115] Phillips M., Childs C., Calder P., Rogers P. (2015). No effect of omega-3 fatty acid supplementation on cognition and mood in individuals with cognitive impairment and probable Alzheimer’s disease: A randomised controlled trial. Int. J. Mol. Sci..

[116] Wagner K.M., McReynolds C.B., Schmidt W.K., Hammock B.D. (2017). Soluble epoxide hydrolase as a therapeutic target for pain, inflammatory and neurodegenerative diseases. Pharmacol. Ther..

[117] Sirish P., Li N., Timofeyev V., Zhang X.-D., Wang L., Yang J., Lee K.S.S., Bettaieb A., Ma S.M., Lee J.H. (2016). Molecular mechanisms and new treatment paradigm for atrial fibrillation. Circ. Arrhythm. Electrophysiol..

[118] Li N., Liu J.-Y., Timofeyev V., Qiu H., Hwang S.H., Tuteja D., Lu L., Yang J., Mochida H., Low R. (2009). Beneficial effects of soluble epoxide hydrolase inhibitors in myocardial infarction model: Insight gained using metabolomic approaches. J. Mol. Cell. Cardiol..

[119] Qiu H., Li N., Liu J.Y., Harris T.R., Hammock B.D., Chiamvimonvat N. (2011). Soluble epoxide hydrolase inhibitors and heart failure. Cardiovasc. Ther..

[120] Wutzler A., Kestler C., Perrot A., Loehr L., Huemer M., Parwani A.S., Attanasio P., Özcelik C., Schunck W.-H., Gollasch M. (2013). Variations in the human soluble epoxide hydrolase gene and recurrence of atrial fibrillation after catheter ablation. Int. J. Cardiol..

[121] Sarkar P., Narayanan J., Harder D.R. (2011). Differential effect of amyloid beta on the cytochrome P450 epoxygenase activity in rat brain. Neuroscience.

[122] Sarkar P., Zaja I., Bienengraeber M., Rarick K.R., Terashvili M., Canfield S., Falck J.R., Harder D.R. (2014). Epoxyeicosatrienoic acids pretreatment improves amyloid β-induced mitochondrial dysfunction in cultured rat hippocampal astrocytes. Am. J. Physiol. Heart Circ. Physiol..

[123] Lee H.-T., Lee K.-I., Chen C.-H., Lee T.-S. (2019). Genetic deletion of soluble epoxide hydrolase delays the progression of Alzheimer’s disease. J. Neuroinflammation.

[124] Griñán-Ferré C., Codony S., Pujol E., Yang J., Leiva R., Escolano C., Puigoriol-Illamola D., Companys-Alemany J., Corpas R., Sanfeliu C. (2019). Pharmacological inhibition of soluble epoxide hydrolase as a new therapy for Alzheimer’s Disease. bioRxiv.

[125] Jacobs V., Woller S.C., Stevens S., May H.T., Bair T.L., Anderson J.L., Crandall B.G., Day J.D., Johanning K., Long Y. (2014). Time outside of therapeutic range in atrial fibrillation patients is associated with long-term risk of dementia. Heart Rhythm.

[126] Jacobs V., May H.T., Bair T.L., Crandall B.G., Cutler M.J., Day J.D., Mallender C., Osborn J.S., Stevens S.M., Weiss J.P. (2016). Long-term population-based cerebral ischemic event and cognitive outcomes of direct oral anticoagulants compared with warfarin among long-term anticoagulated patients for atrial fibrillation. J. Am. Coll. Cardiol..

[127] Bunch T.J., Crandall B.G., Weiss J.P., May H.T., Bair T.L., Osborn J.S., Anderson J.L., Muhlestein J.B., Horne B.D., Lappe D.L. (2011). Patients treated with catheter ablation for atrial fibrillation have long-term rates of death, stroke, and dementia similar to patients without atrial fibrillation. J. Cardiovasc. Electrophysiol..

[128] Bordier P., Lanusse S., Garrigue S., Reynard C., Robert F., Gencel L., Lafitte A. (2005). Causes of syncope in patients with Alzheimer’s disease treated with donepezil. Drugs Aging.

[129] Malone D.M., Lindesay J. (2007). Cholinesterase inhibitors and cardiovascular disease: A survey of old age psychiatrists’ practice. Age Ageing.

[130] Lin Y.-T., Wu P.-H., Chen C.-S., Yang Y.-H., Yang Y.-H. (2016). Association between acetylcholinesterase inhibitors and risk of stroke in patients with dementia. Sci. Rep..

